# Experiences of working from home: umbrella review

**DOI:** 10.1093/joccuh/uiad013

**Published:** 2023-12-14

**Authors:** Charlotte E. Hall, Samantha K. Brooks, Freya Mills, Neil Greenberg, Dale Weston

**Affiliations:** Department of Psychological Medicine, King’s College London, Weston Education Centre, London, SE5 9RJ, United Kingdom; Behavioural Science and Insights Unit, Evaluation & Translation Directorate, Science Group, UKHSA, Porton Down, Salisbury, SP4 0JG, United Kingdom; Department of Psychological Medicine, King’s College London, Weston Education Centre, London, SE5 9RJ, United Kingdom; Behavioural Science and Insights Unit, Evaluation & Translation Directorate, Science Group, UKHSA, Porton Down, Salisbury, SP4 0JG, United Kingdom; School of Psychology, University of Sussex, Brighton, BN1 9QH, United Kingdom; Department of Psychological Medicine, King’s College London, Weston Education Centre, London, SE5 9RJ, United Kingdom; Behavioural Science and Insights Unit, Evaluation & Translation Directorate, Science Group, UKHSA, Porton Down, Salisbury, SP4 0JG, United Kingdom

**Keywords:** umbrella review, working from home, WFH, experience of homeworking

## Abstract

Introduction: The concept of “working from home” is extremely topical following the COVID-19 pandemic; therefore, it is unsurprising that there has been an increased interest in collating research related to homeworking. This has been carried out by multiple reviews, all with slightly different research aims and methodologies. Collating the findings from the available reviews is therefore highly beneficial to establish the experience of homeworking to create recommendations for the future of home-based work.

Methods: An umbrella review was carried out. In June 2022, literature searches were conducted across 4 electronic databases. Published reviews of literature that used a systematic process, were focused on working from home populations, and detailed factors that could be related to the personal experience of homeworking (eg, barriers, facilitators, advantages, disadvantages) were included.

Results: A total of 1930 records were screened and 6 review articles were included. Results report on the following sections: working environment (eg, workplace design, space conditions), personal impact (eg, satisfaction, career impact), and health (eg, physical health, well-being) including a total of 19 themes. Mixed findings were apparent for nearly all included themes, highlighting the need to consider individual and contextual circumstances when researching working from home.

Conclusions: This review establishes the importance of retaining flexibility while homeworking for employees, managers, and organizations. Essentially, a one-size-fits-all approach to working from home is impractical as individual circumstances limit application. Eight recommendations for the future of working from home are suggested.

## Teaser

**Key points:** Working from home as a result of the COVID-19 pandemic saw a vast number of individuals adapt to a new way of working, and much research has sought to establish the impact of this change.

This umbrella review collates and synthesizes the findings of 6 systematic-style reviews focused on homeworking literature. Nineteen themes were extracted from the data covering the categories of working environment, personal impact, and health. Nearly all included themes reported mixed literature.

The results highlight the importance of retaining flexibility whilst homeworking for employees, managers, and organizations. Individual circumstances and personal contexts mean that a “one-size-fits-all” approach to the management of working from home is not fit for purpose. Recommendations about the future of working from home are discussed.

## 1. Introduction

The coronavirus (COVID-19) pandemic saw the introduction of several behavioral interventions aimed to reduce the transmission of the virus (eg, social distancing, lockdowns, and self-testing). A major change experienced across many countries was the transition to “work from home.” For example, in the United Kingdom, the Prime Minister made a statement^1^ on March 16, 2020 asking the public to work from home if possible, and as a result nearly half of those in employment reported to work from home in April 2020.^2^ Working from home (WFH) was also reported in half of the US workforce in May 2020^3^ and across the European Union in July 2020.^4^

Prior to the pandemic, literature provided evidence of a mixed impact of homeworking in terms of benefits and consequences associated with WFH. For example, eliminating commuting time is seen as a benefit of WFH,^5^ but negative consequences such as difficulties establishing boundaries between work and home life have also been reported.^6^ As a result of sudden changes to working style brought by the COVID-19 pandemic, there has been an influx in research surrounding WFH and related constructs, often commonly referred to, and often used interchangeably in the literature, as working from home (eg,^7^), telework (eg,^8^), and telecommuting (eg,^9^).

Given the concept of “working from home” is extremely topical following the COVID-19 pandemic, a cursory examination of the literature suggested that it would be highly beneficial to collate findings from literature reviews examining WFH and related constructs in order to establish recommendations for the future of home-based work. In order to do this, a review of reviews, known as an umbrella review^10^ methodology, was chosen and is believed to be the first in the area. The current umbrella review sought to: (1) describe the WFH experience documented across the literature, to aid in identifying common factors that may make WFH easier, or more difficult, for individuals; (2) establish gaps in the literature in order to inform future research; and (3) create a series of recommendations for the future of home-based work.

## 2. Methods

The current umbrella review followed the Preferred Reporting Items for Systematic Reviews and Meta-Analyses (PRISMA) guidelines.^11^ The following sections detail: the search strategy, eligibility criteria, review selection, quality assessment, data extraction, and data synthesis.

### 2.1. Search strategy

The search was conducted by the first author on June 15, 2022 across the following databases: Ovid®SP MEDLINE® 1946 to June 15, 2022; Ovid®SP Embase 1974 to June 15, 2022; Ovid®SP APA PsycINFO 1806 to June week 3, 2022; Web of Science™ Core Collection. The search involved 2 strings of terms: firstly, those relating to homeworking, and secondly, those relating to barriers, facilitators, advantages, or disadvantages.

**Figure 1:**
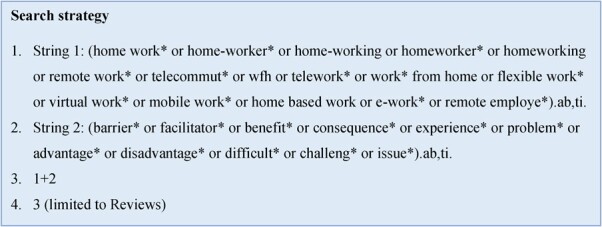
Search strategy used in experiences of working from home: umbrella review.

The search terms were developed a priori from current literature, iteratively alongside preliminary searches, and through research team meetings to ensure a manageable and focused scope of investigation. The final search terms used are presented in Figure 1, and full search strategies for all databases, including filters and limits used can be found in Table S1.

### 2.2. Eligibility criteria

The development of inclusion and exclusion criteria for the current review was iterative and developed alongside literature familiarization, preliminary database searches, and research team meetings. To be included in this umbrella review literature reviews must: (1) have been carried out in a systematic way (ie, included a search strategy, and detailed how many papers are used within the synthesis of results) as this allows for comparison and contrast of high-level evidence^10^; (2) focus on a population of adult desk-based homeworking individuals; and (3) detail personal factors associated with WFH, or related constructs (ie, telecommuting, teleworking), in relation to personal advantages, disadvantages, barriers, or facilitators to/of homeworking, as per the aims of this review. The final detailed inclusion and exclusion criteria for the current umbrella review can be found in Table 1.

**Table 1:** Inclusion and exclusion criteria.

**Category**	**Inclusion**	**Exclusion**
	Published reviews (systematic, scoping, rapid) and meta-analysis	Primary research and gray literature
	Reviews that contain a search strategy and detail how many papers are used in the analysis/synthesis	Reviews that do not contain a search strategy or do not detail how many papers are used in the analysis/synthesis
	Published in English	Not available in English
**Type of study**	Full text available	No full text available
	Includes studies that focus on the homeworking population	Includes studies that focus on populations who are unable to work from home
	Includes studies that focus on homeworkers who work from a desk, in a non-manual job	Studies that focus on populations who have manual jobs (eg, live-in carers or nurses)
**Population/context**	Includes studies that focus on working adults	Studies with a focus on populations drawn from the education setting (eg, online students, university students)
**Factor(s)**	The review details personal factors associated with working from home in relation to personal advantages, disadvantages, barriers, or facilitators to/of homeworking	The review does not detail any factors associated with working from home in relation to personal advantages, disadvantages, barriers or facilitators to/of homeworking, or details only factors relating to business or environment (eg, carbon footprint, climate change)

### 2.3. Review selection

Search results were loaded into Endnote bibliographic software and deduplicated. Title and abstract screening were carried out on all reviews by one reviewer (C.E.H.), with each being assessed using the inclusion and exclusion criteria and filtered out at this stage if deemed non-relevant. All retained reviews were then full-text screened by C.E.H., with a second reviewer (F.M.) screening an additional 20% of the records to provide robustness to the process. Any disagreements were discussed between reviewers and a collaborative decision made. Reviews were then marked as “include” or “exclude.” Throughout the screening process there were regular research team meetings to discuss progress and any difficulties. A PRISMA^11^ flow diagram of study selection was used to display the process.

**Figure 2:**
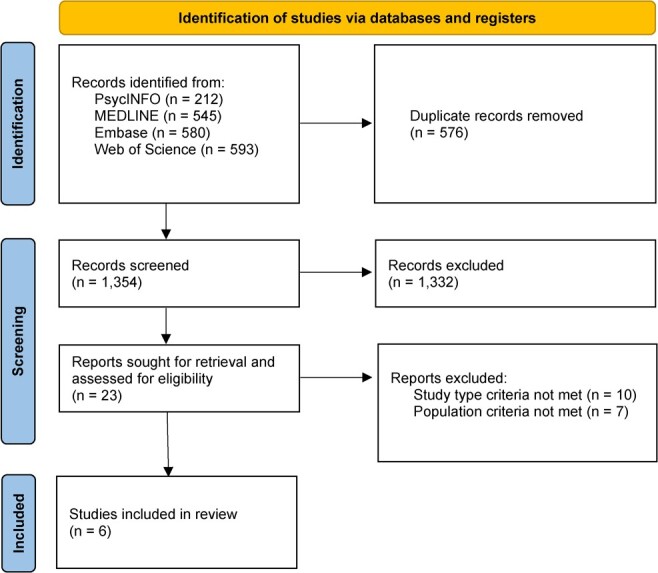
PRISMA flow diagram.

### 2.4. Quality assessment

The quality of each of the included reviews was rated using the Assessing the Methodological Quality of Systematic Reviews AMSTAR-2: a critical appraisal tool for systematic reviews.^12^ The AMSTAR-2^12^ is a 16-item assessment tool that assesses systematic reviews on various domains, including: design, search strategy, screening process, description of included studies, and assessment of risk of bias. The AMSTAR-2 is not intended to be scored, and instead includes 7 “critical” criteria and 9 “standard” criteria. Interpretation of the assessment results in reviews being classed as “high” (ie, 0 or 1 non-critical weakness), “moderate” (ie, more than 1 non-critical weakness), “low” (ie, 1 critical flaw with or without non-critical weaknesses), or “critically low” (ie, more than 1 critical flaw with or without non-critical weaknesses) quality. More details can be found in Table S2.

### 2.5. Data extraction and synthesis

Article data and information extracted included: authors; title; type of review; publication year; first author’s country of origin; number of papers included within the review and their citations; search strategy; database(s) searched; quality appraisal tool(s) used; review funding details; sample demographics and characteristics; key results; and implications and recommendations. Relevant extracted data from the included reviews’ results were coded in themes using thematic analysis^13^; this was carried out by coding the collected data and then grouping to allow for comparison (eg, data regarding pain, discomfort, or injury were grouped together). Due to a large number of themes being apparent in the data, the individual themes (eg, workplace design, satisfaction) were then further categorized at a higher level of abstraction to allow the results to be appropriately structured so a coherent and logical analytic narrative could be developed.^13^ The 3 categories used were: “working environment,” “personal impact,” and “health.” With all data themed and categorized, a narrative was then formed.^14^

## 3. Results

A total of 1930 records were identified through literature database searching. Following the removal of duplicates, 1354 records remained and were then title and abstract screened. Twenty-three records were sought for retrieval and obtained. Following full-text screening, 6 reviews remained.^15^^–^^20^ A PRISMA flow diagram, including the numbers for inclusion and exclusion at each stage of the current review, is presented in Figure 2. Studies excluded at the full-text screening stage are also described in Table 2.

**Table 2:** Articles excluded at the full-text screening stage.

**Article reference**	**Reason**
Alaiad, A, Y Alnsour, and M Alsharo, Virtual teams: thematic taxonomy, constructs model, and future research directions. *IEEE Transactions on Professional Communication*, 2019. 62(3): p. 211-238.	Falls on population/context
Allen, TD, TD Golden, and KM Shockley, How effective is telecommuting? Assessing the status of our scientific findings. *Psychological Science in the Public Interest*, 2015. 16(2): p. 40-68.	Falls on type of study
Ammons, SK and WT Markham, Working at home: experiences of skilled white collar workers. *Sociological Spectrum*, 2004. 24(2): p. 191-238.	Falls on type of study
Anka, A, H Thacker, and B Penhale, Safeguarding adults practice and remote working in the COVID-19 era: challenges and opportunities. *Journal of Adult Protection*, 2020. 22(6): p. 415-427.	Falls on population/context
Appleton, R, J Williams, NVS Juan, et al, Implementation, adoption, and perceptions of telemental health during the COVID-19 pandemic: systematic review. *Journal of Medical Internet Research*, 2021. 23(12) (no pagination)(e31746).	Falls on population/context
Arunprasad, P, C Dey, F Jebli, A Manimuthu, and Z El Hathat, Exploring the remote work challenges in the era of COVID-19 pandemic: review and application model. *Benchmarking-an International Journal*.	Falls on type of study
Bandyopadhyay, SK, V Goyal, and S Dutta, Problems and solutions due to mental anxiety of IT professionals work at home during COVID-19. *Psychiatria Danubina*, 2020. 32(3-4): p. 604-605.	Falls on type of study
Beckel, JLO and GG Fisher, Telework and worker health and well-being: a review and recommendations for research and practice. *International Journal of Environmental Research and Public Health*, 2022. 19(7).	Falls on type of study
Birimoglu Okuyan, C and MA Begen, Working from home during the COVID-19 pandemic, its effects on health, and recommendations: the pandemic and beyond. *Perspectives in Psychiatric Care*, 2022. 58(1): p. 173-179.	Falls on type of study
Boavida, N and AB Moniz, Virtual work in Portugal: a literature review. *International Journal on Working Conditions*, 2020(19): p. 1-15.	Falls on type of study
Bolino, MC, TK Kelemen, and SH Matthews, Working 9-to-5? A review of research on nonstandard work schedules. *Journal of Organizational Behavior*, 2021. 42(2): p. 188-211.	Falls on population/context
Crawford, JO, L MacCalman, and CA Jackson, The health and well-being of remote and mobile workers. *Occupational Medicine-Oxford*, 2011. 61(6): p. 385-394.	Falls on population/context
Garcia, LME, HV Guerrero, and AJ Rodriguez, Safety and health conditions at work of teleworkers: systematic review. *Revista Pensamiento Americano*, 2019. 12(23): p. 94-104.	Falls on type of study
Johnson, A, S Dey, H Nguyen, et al, A review and agenda for examining how technology-driven changes at work will impact workplace mental health and employee well-being. *Australian Journal of Management*, 2020. 45(3): p. 402-424.	Falls on type of study
Liu, W, Y Xu, and D Ma, Work-related mental health under COVID-19 restrictions: a mini literature review. *Frontiers in Public Health*, 2021. 9: p. 788370.	Falls on type of study
Shiri, R, J Turunen, J Kausto, et al., The effect of employee-oriented flexible work on mental health: a systematic review. *Healthcare*, 2022. 10(5): p. 10.	Falls on population/context
Stich, JF, A review of workplace stress in the virtual office. *Intelligent Buildings International*, 2020. 12(3): p. 208-220.	Falls on population/context

### 3.1. Article characteristics

In total, 6 reviews met the inclusion criteria and were used within this analysis. Review articles originated from Brazil,^16^ Greece,^15^ Switzerland,^20^ Norway,^18^ Italy,^17^ and Australia,^19^ and were published between 2020 and 2022. The reviews’ inclusion criteria for data differed: 1 review allowed inclusion of papers published between 2000 and 2020^15^; 1 allowed inclusion of papers published between 1990 and 2020^20^; 1 review excluded papers with data collected prior to 2007^19^; 1 review excluded papers published prior to 2011^17^; 1 had no date restriction, but literature searches were carried out in 2018^16^; lastly, 1 review included papers published from 2010 to 2020, but excluded studies where there were strict restrictions due to COVID-19, as it was believed that this would bias the effects of WFH.^18^ The number of studies included within these reviews ranged from 12 to 70. Further review characteristics can be found in Table S3. To allow for the examination of recurring papers throughout the reviews, citation lists for each of the 6 reviews were extracted and compared. On average, the reviews cited 81% unique references. The AMSTAR-2 rated 3 reviews to be “high” quality, 1 “low” quality, and 2 “critically low.” Both “critically low” quality reviews did not assess for risk of bias in individual studies included in their review, nor could they account for risk of bias in individual studies when interpreting or discussing the results of the review. Additionally, none of the 6 included reviews reported on the sources of funding for the studies included in their review. Due to the small number of reviews meeting the inclusion criteria, none of the reviews were removed post quality appraisal.

There were many different themes presented across the 6 systematic reviews. Therefore, results have been thematically analyzed into the following sections: working environment, personal impact, and health. For clarity, the number of supporting citations for findings within the reviews are listed in parentheses where appropiate.

### 3.2. Working environment

*Workplace design:* One review^20^ reporting on workplace design contained 3 studies where home-based teleworkers reported having uncomfortable furniture, lack of storage for devices and office supplies, and poor lighting conditions. Issues such as: weak screen positioning and small desktop areas; no foot contact with the ground; and insufficient backrest support for the spine, were also reported.

*Availability of workspace:* One review^20^ reported on space conditions whilst homeworking. Two of the included studies found that some teleworkers work from 1 dedicated room, and another study concluded that 70% of teleworkers use living rooms or bedrooms to work from. Furthermore, the review reported that teleworkers often choose the smallest or least contested space available in their house to avoid inconveniencing family members.^20^ Furthermore, 1 cited study stated the importance of working from a separate room to control boundaries with family members.^20^ Background noise and distractions whilst home working were also noted as a disruption.^20^

*Training:* One review^20^ reported on training for teleworkers. Two cited studies found that most teleworkers receive no training, with another reporting that fewer than 50% of teleworkers receive training for specific tasks, with even less having training on lifestyle changes associated with homeworking.

*Taking breaks:* Two reviews^17^^,^^20^ reported on use of technology. The first^20^ contained 4 studies where home-based teleworkers reported working longer periods at home without taking a break, as well as working on weekends and evenings, essentially more than they would whilst in the workplace. Time spent on breaks, and the time spent working at a computer without breaks, were both reported to be shorter at home than in the office. The time spent on the telephone was also double the duration at home compared with office working. The second review^17^ reported WFH was associated with more screen time per day compared with those without employment change (1 citation).

*Autonomy:* Two reviews^15^^,^^16^ examined autonomy and presented mixed findings. Findings established that teleworking results in a reduction of direct supervision, which results in more autonomy, increased responsibility, and further opportunities to demonstrate performance and value.^16^ Another review^15^ found the flexibility associated with WFH to be beneficial for those with children (1 citation) and international employees (1 citation). Additionally, working in a teleworking environment was found to lead to positive emotions due to perceived autonomy, control, and flexibility (1 citation). However, establishing a balance between autonomy and control to ensure flexibility whilst also ensuring efficient working (3 citations) was noted to be of importance whilst WFH.^15^ Other cited research suggests it may also result in intensification of work (1 citation).^15^

### 3.3. Personal impact

*Productivity:* Two reviews^15^^,^^16^ examined productivity and presented mixed findings. WFH was generally claimed to improve productivity (4 citations), with additional factors (ie, implementation processes (1 citation) and human resources practices (2 citations) moderating the relationship.^15^ Additional research reported that teleworking does result in work intensifying, but is carried out in a more pleasant environment, which in turn increases productivity.^16^ However, the teleworking environment may positively affect productivity of creative tasks but negatively impact productivity of tasks perceived as dull.^15^ Furthermore, 1 cited study found telework can impact negatively on productivity when the worker has additional non-work commitments (eg, raising children).^15^

*Satisfaction:* Two reviews^15^^,^^18^ examined satisfaction, one in the form of life and leisure and the other in terms of job satisfaction, and presented mixed findings. The first review included 2 studies; one found no evidence for a relationship between WFH and leisure satisfaction, whereas the other found an association between WFH and leisure satisfaction, but not with overall life satisfaction.^18^ The second review established that there are many supporting studies for a positive relationship between teleworkers and increased job satisfaction, “especially under specific circumstances” (p. 12; 7 citations).^15^ However, there was also a study cited that reported equally high levels of job satisfaction between both office and home workers.^15^ Additionally, other cited research suggests that a curvilinear relationship is apparent between the extent of job satisfaction and telework, which reaches a point where the time spent teleworking is no longer associated with job satisfaction.^15^

*Career prospects:* Two reviews^15^^,^^16^ examined the impact of home-based work on career prospects and presented mixed findings. Two of the cited references associated home-based working with stunted career progression, with a further study finding that teleworking employees fear homeworking may reduce opportunities for advancing their career.^15^ It was theorized that telework may be associated with negative career outcomes due to a perceived lack of dedication (1 citation), supported by 1 citation that found that women who telework are perceived by others to be not working (no detail as to who “others” are was provided in the review^15^). Another cited study established that there is no difference between those who work from home versus those who work from the office in terms of promotions, but those who telework have less salary growth.^15^ However, the second review established that teleworking reinforces the self-image of responsible, committed, independent, and autonomous professionals, as well as providing opportunities for younger employees to show professional maturity.^16^

*Work-life balance:* Three reviews examined work-life balance^15^^–^^17^ and presented a mixed literature. In the first review, 7 papers were referenced that supported the finding that telework is associated with high levels of work-life balance.^15^ However, several factors were reported to influence this relationship, such as institutional setting of each country (ie, who has access for flexible working; 2 studies), boundaries between work and home life (4 citations), and autonomy (2 citations).^15^ The second review^17^ found WFH was beneficial for reducing work–family conflict when WFH days alternated with office days (1 citation). The third review established that flexible working hours and avoiding commuting have a positive influence on the balance between family and work life.^16^ However, the same review also remarked that conflict between work and family life was one of the most prominently cited disadvantages across all included papers in their review.^16^

*Isolation:* Two reviews examined social isolation^15^^,^^16^ and presented mainly negative findings. In general, WFH may negatively impact social and professional interaction and result in isolation (4 citations).^15^ Similarly potential exclusion of those WFH can result in loneliness, disconnection, and negative emotions (3 citations).^15^ Other cited research suggests that it depends on the individual as to whether homeworking will result in isolation (ie, how much of a social life is needed, individual characteristics, or attitude towards technology; 3 citations).^15^ Literature presented in this review also establishes that part-time teleworking can alleviate the negative effects of a lack of interaction.^15^ The second review found that isolation was one of the most prominently cited disadvantages across all included papers in their review.^16^

*Working relationships:* One review examined the impact of homeworking on working relationships.^15^ This review claimed that teleworkers potentially experience social and professional exclusion alongside loneliness and disconnection (3 citations). Teleworkers were also found to fear that those who did not telework would doubt their commitment and contribution in the workplace (1 citation). Cited studies found: face-to-face contact mediates a relationship between trust and worker type (ie, teleworkers vs office workers); knowledge transfer is negatively affected by homeworking (1 citation); superficial connections and fears of inability to cooperate result in teleworkers wishing to work from the workplace on days they were teleworking (1 citation).^15^

### 3.4. Health

*General health (including healthy behaviors):* Five reviews reported on general health and healthy behaviors^16^^–^^20^ and presented mixed findings. One review contained a study that found that those WFH experienced less risk of developing poor health (based on underlying health risk factors, measured by Edington score) compared with office workers,^18^ but also included 2 studies that did not find any significant relationship between part- or full-time WFH and self-reported general health.^18^ A second review contained a study that reported that teleworkers believed themselves to be more “fit to work” at home compared with when they worked in an office; this was also true for those with chronic illnesses.^20^ Better eating behaviors (compared with coworking spaces) were also reported (1 citation).^20^ The third review^17^ found the transition to homeworking during the COVID-19 pandemic was also associated with an increase in intake of vegetables, fruit, dairy, snacks, and self-made meals; younger workers and females benefitted the most in terms of healthier eating (1 citation).^17^WFH was also reported to be associated with increased alcohol and tobacco consumption (1 citation). Weight gain was reported in around half (46.9%) of a study population of employees who transitioned to WFH due to COVID-19 (1 citation). This was also supported by another study that established a 41% prevalence of weight gain in 869 homeworkers during the COVID-19 pandemic (1 citation). The fourth review^17^ concluded that part-time telecommuters experience better emotional and motivational outcomes on homeworking days compared with office working days.^16^ The fifth review^19^ reported that government employees were found to feel “safer working from home” (1 citation) and this was associated with increased energy levels (1 citation). Another included a study of employees who were given the opportunity to work from home part-time; they found a small but statistically significant decrease in self-reported health in those WFH compared with a control group.

*Physical health:* Two reviews reported on physical health^17^^,^^20^ and presented mixed findings. One review^17^ found those WFH spent significantly less time engaging in physical activity compared with those not WFH. The same research also found more uninterrupted sedentary behavior was reported in those WFH compared with those not WFH (1 citation). Similarly, 1 cited study in the same review found that both WFH and lost employment were associated with longer time spent in sedentary behavior compared with those who did not change employment status during the COVID-19 pandemic (1 citation). A second review^20^ found that physical activity was not positively or negatively impacted by WFH, but sedentary behavior did increase (1 citation). Conversely, more physical activity, compared with coworking spaces, was also reported by one cited study.^20^

*Pain and discomfort:* Two reviews reported on pain and discomfort in those WFH^18^^,^^20^ and presented mainly negative findings. One review reported 2 cross-sectional studies relating to general pain: one found no association between WFH (on normal hours across weekdays) and pain, whereas the other found that males WFH reported significantly lower levels of pain (not found in females).^18^ Another review^20^ reported the following findings in relation to pain and discomfort: pain in upper limbs, back, and neck attributed to inadequate furnishings (1 citation); eye strain and wrist pain for those who used laptops (1 citation); migraines, eye strain, shoulder tendinitis, sore back, neck pains, and wrist pain from working in home offices (1 citation); and 43% of teleworkers experiencing musculoskeletal issues. Following medical examination, 12% of these musculoskeletal issues were found to be work-related complications, such as cervical spine symptoms and muscular tension (1 citation); stiff shoulders, eye strain, and lower back pain were common complaints that impacted job performance and led to ongoing treatment (1 citation). Two studies reported that home-based teleworkers experienced discomfort whilst working, and the longer they worked, the higher the incidence of work-related discomfort. This review also reported findings relating to long-term effects of WFH arising from pain and discomfort.^20^ One study reported that homeworkers believed they would experience computer-related health problems in the next 5 years (1 citation), and 2 studies reported that WFH-related injuries resulted in work absence (ie, musculoskeletal injuries resulting in 80 days of absence, shoulder tendonitis resulting in 45.2 days of absence, tension neck syndrome resulting in 4.6 days of absence).

*Presenteeism:* One review examined presenteeism.^20^ Within the review, 1 study suggested that teleworkers may ignore symptoms of illness, with many still working when they are ill (1 citation). Home-based workers were also reported to not always prioritize their own health because of the advantages associated with homeworking (eg, opportunities to provide childcare, elimination of emotional stress; 4 citations). Additionally, a second study found that the health risks and experiences of WFH are trivial compared with the stress they experienced within the office (1 citation).

*Well-being:* Two reviews reported on well-being^18^^,^^19^ and presented mixed findings. The first review^18^ found both positive and negative affective well-being were influenced positively when employees were WFH compared with when working at the office (1 citation). WFH benefitted early mothers in terms of fewer depressive symptoms (1 citation), and associations were found between WFH and experiencing happiness, sadness, and meaningfulness, but a small reduction in mothers was also shown (1 citation). Lastly, 1 study reported no relationship between WFH and happiness or sadness. The second review^19^ found a full-time WFH “pilot” carried out over 3 months that resulted in employees reporting an improved sense of well-being (1 citation). Increased well-being was also found in a sample of government employees (1 citation), as well as those in the education and private sectors (1 citation) and university employees (1 citation). Communication and support from colleagues were found to influence psychological well-being for those WFH (1 citation). Additionally, participants had a preference for hybrid working to allow connection with work colleagues (1 citation).

*Mental health:* Two reviews^17^^,^^19^ reported on mental health concerns and presented mixed findings. One cited study established that lower levels of physical activity, poor sleep quality, being female, and long working hours during the pandemic were associated with a greater risk of depression among remote workers. Another cited study also found that WFH is a predictor of increased depressive symptoms. Conversely, the second review^17^ established that those WFH for less than 8 hours per month had statistically lower levels of depression than those not WFH.^17^

*Stress:* Four reviews reported on stress^17^^–^^20^ and presented mixed findings. The first review^18^ included 3 studies that reported a relationship between WFH and reduced stress levels (although this may depend on gender and the number of dependents in the home), whereas 1 study found an association with higher stress, and another found no relationship at all; part-time WFH was found to have less stressful WFH days, but there was no difference in stress level between those teleworking partly at home and those working only at the office (1 citation).^18^ The second review established that blood pressure (indicative of stress) was higher at company offices, and those who worked from home scored mood and calmness more positively than when they worked from the office.^20^ The third review^17^ reported that in a sample of academics provided the choice to work from home, greater relaxation (as measured by heart rate variability) was found to occur whilst homeworking (1 citation). Females and those with lower incomes (1 citation), as well as those who live alone (1 citation) were more likely to experience stress whilst WFH. Stress was also found to be associated with family conflict and social isolation whilst WFH during the pandemic (1 citation). The fourth review^19^ cited 2 studies that suggested that working more hours from home was associated with less strain (1 citation) and less emotional exhaustion and cognitive stress (1 citation). Conversely, 1 cited study found WFH was associated with increased levels of exhaustion when combined with a high level of work/family conflict (1 citation). A decrease in stress levels of employees homeworking part time was reported by 5 cited studies, and in employees who had additional caring responsibilities (1 citation). Other research suggested no relationship (1 citation), or a positive relationship (1 citation) between stress and homeworking.

**Table 3:** Recommendations suggested by retained reviews.

**Grouping**	**Recommendation**	**Reference**
	Training on establishing a home office for employees	20
	Training on home-based policies, ergonomics programs, and health-related consequences associated with the absence of ergonomic support	20
	Educate workers on a healthy lifestyle in the working from home setting	17
	Training in necessary software and systems needed by an individual to work from home	19
	Organizations need to provide training and assistance for managers supervising work from home staff	19
	Educate workers on how to develop boundaries more formally between work and family	19
	Managers should be made aware of the challenges stemming from the implementation of telework (eg, requiring new methods of managerial control to be adopted and tested)	15
**Education/training**	Managers and employees (regardless of working arrangement) should be prepared for the risks involved with telework (eg, tensions in working relationships), and be informed of ways to overcome any difficulties (eg, certain human resources practice)	15
	Financial compensation for employees working from home should be considered	19, 20
	Provision of appropiate equipment should be ensured	19
	To aid career growth concerns, employers and employees should choose or adjust the most appropriate teleworking plan based on their need for professional growth and productivity	15
	Ensuring there is regular communication to staff around role expectations, performance measures, workloads, and access to human resources support	19
	Ensure those who choose or are mandated to work at home do not experience negative career consequences	19
	Systems that optimize regular, reliable, and consistent communication using methods that are appropriate for employers and employees need to be implemented	19
	A key policy to support working from home should be targeted at the development of adaptable strategies to ensure they meet the nuanced needs of different employees, irrespective of gender or life course stage	19
	Working arrangements should be optimized by employers to potentially reduce stress and exhaustion and increase the feeling of well-being in employees	18
	Human resources practices could be used to reverse feelings of isolation	15
**Practical**	In situations where working from home is voluntary, employees may benefit from a regular day in the office to maintain social networks. In situations where working from home is mandatory, provision of regular face-face online contact opportunities and social support could replace the day in the office	19

*Exhaustion and burnout:* Two reviews reported on exhaustion and burnout^18^^,^^19^ and presented mixed findings. The first review cited research that found that WFH led to a lower degree of exhaustion but could be mediated by multiple factors (eg, time pressure, support, role conflict; 1 citation); part-time WFH could have mixed effects on exhaustion (1 citation); males experienced reduced tiredness when homeworking (1 citation); and negative experiences whilst WFH were associated with burnout (1 citation). The review also cited research suggesting that there was no association between WFH and tiredness (1 citation) or emotional exhaustion (1 citation).^18^ The second review^19^ found homeworking was associated with decreased fatigue levels (2 citations), and mixed findings where a cited study reported that the negative effects did not outweigh the overall positive impact of WFH (1 citation). Others found WFH had no impact on exhaustion (2 citations).

### 3.5. Recommendations and future research

Recommendations for the future of home-based work or future research suggestions relating to homeworking presented by the included reviews can be found in Tables 3 and4. Training needs for staff included: how to establish a home office and health-related consequences of lacking ergonomic support^20^; how to establish boundaries between work and home life^19^; relevant training in software and systems for working^19^; and how to maintain a healthy lifestyle whilst homeworking.^17^ Training needs for managers included: specialized training for managing staff who are working from home^19^; and training on awareness of telework challenges.^15^ Practical recommendations included: considering financial compensation for staff^19^^,^^20^; ensuring provision of appropriate equipment^19^; ensuring career progress is not impacted by working from home^15^^,^^19^; good communication^19^; social opportunities^19^; and, optimized working arrangements to increase well-being.^18^

**Table 4:** Future research suggestions from retained reviews.

**Grouping**	**Research recommendation**	**Reference**
	Ergonomics, ergonomic interventions, and work-related disorders	20
	Virtual ergonomics programs and integration into daily life	20
	Enablers and constraints to teleworking	15
	How to reduce the health risks and increase the benefits of working from home	20
	Leadership style as an enabler or constraint	15
	The potential of teleworking for employees with disabilities	20
	Differences in economies or technological development of the countries where teleworking is taking place	15
**Research topics**	Technostress and other psychosocial effects of using information and communications technology in the “new normal”	15
	Working from home and social interactions	15
	Working from home and coexistence of at least 2 teleworkers in the same house	15
	Working from home and gender, family situation, and type of job	18
	Working from home and all factors in the relationship between employees and their organizations	19
	Intensity of telework and potential moderators of this relationship (eg, optional vs compulsory, tasks carried out)	15
**Impact**	Impact of teleworking in other areas of life (eg, health, job satisfaction, performance, cost reduction for workers/employers) whilst considering personal, business, and social level spheres	16
	Interventions with random allocation	18
**Research methodology**	Longitudinal research needed	19

## 4. Discussion and recommendations

The current umbrella review sought to collate findings from existing reviews to describe the WFH experience and establish common barriers and facilitators to WFH. Specifically, this review synthesised 6 peer-reviewed review articles and established findings in relation to working environment, personal impact, and health covering a total of 19 themes. Despite generally mixed literature, this synthesis has allowed for the development of 8 key recommendations and guidelines for future WFH arrangements, which are discussed below.

In relation to working environment: working using inadequate or unergonomic furniture (eg, small desktop areas, insufficient backrest support); potentially working longer periods without taking breaks; working in a small undedicated makeshift area (eg, in a bedroom or living room); and lack of training in homeworking were all potential barriers or areas that could be improved in the future of WFH. The literature in relation to pain and musculoskeletal issues was mixed, but presented mainly negative findings, as retained reviews included some cited research that stated that pain was reduced whilst WFH (specifically for males^18^), and others that experiencing pain and discomfort were associated with homeworking and could result in long-term effects and work absence.^20^ As a result, it is recommended that employers should ensure that staff have the right equipment and training to work safely and comfortably from home. Whereas workplace safety and comfort can be easily appraised in the office, through in-person workstation assessments (eg,^21^), the safety of home offices is less easy to appraise. This could be achieved by developing online assessments of workplace ergonomics, where employees would complete a survey to assess whether their home set-up is appropriate or not. Where problems are identified, tips for rectifying these problems could be provided as part of the results of the assessment, and where necessary organizations should provide additional equipment if it is financially viable to do so. Additionally, employers and employees should recognize that taking regular breaks from work is helpful. Allied to ergonomic workstations, this has the potential to result in reduced physical health problems. Ensuring employees are taking breaks is more difficult whilst not working in a shared space. To overcome this, employers should be proactive in sending regular reminders (eg, using emails, staff newsletters, informal chats with managers) for employees to take regular breaks. Managers could also provide information justifying the need for this and describing the benefits of taking regular breaks to staff. These recommendations are supported by additional literature suggesting that employers may not realize that not all employees have access to well-suited home working set-ups, and recommending that funding and time should be invested into ensuring staff are WFH safely^22^; it is also suggested that staff should be proactive in requesting resources and materials needed to work from home.^22^

The current review indicated that those WFH may engage in presenteeism. A systematic review examining the impact of presenteeism on health and well-being established that sickness presenteeism is associated with future poor self-rated health and sickness absence.^23^ Working whilst ill has also been found to increase risk of depression,^23^ and reduce productivity.^24^ The findings from the current review indicate workers do not prioritize their own health, with 1 retained review^20^ suggesting it is due to WFH being so beneficial, and that the experiences/risks of WFH are perceived to be so trivial compared with office working, that staff continue to work despite being ill. Therefore, it is recommended that managers should be setting examples and acting as role models for healthy behavior. All employees should view home working just as they would office working and take regular breaks, taking leave, including sickness leave when required, and avoiding extensive sedentary behavior. To do this effectively, managers will have to be proactive in displaying good behavior to colleagues and finding ways to communicate this in the WFH context. For example, regular catch-ups with employees in which they make clear that they themselves are taking breaks, are booking and taking leave, and only working within their scheduled hours unless otherwise arranged.

Factors related to mental health and well-being (ie, exhaustion and burnout, mental health, well-being, and stress) all had mixed findings presented in the included reviews. Additional review literature relating to mental health and homeworking, using non-systematic review methods, were also found in the search for the current review and concluded mixed findings (eg,^25^). As a result, it is surmised that the relationship between mental health and homeworking is complex, with the potential to be impacted by many personal contextual factors (eg, gender, number of dependents at home, work demands).^26^ Therefore, it is recommended that staff asked to WFH should be provided with adequate resources and guidance about how to maintain their mental health and psychological resilience. To do this, homeworkers would require tailored guidance specifically about WFH. This could include acknowledgment of benefits and challenges associated with homeworking, and emphasizing maintaining healthy behaviors (eg, taking breaks) whilst WFH.

Findings relating to isolation whilst home working were mixed, essentially depending on each worker’s individual preferences and working style (eg, how much of a social life is needed, individual characteristics, or attitude towards technology). Working relationships were also found to be negatively impacted by homeworking, with superficial connections with colleagues, workplace exclusion, and concerns about commitment to work being some of the key reasons for this impact. Wider occupational literature also states the importance of work/team level social support on productivity (eg,^27^) and work-life balance (eg,^28^^,^^29^). As a result, it is recommended that line managers and employers should be proactive in encouraging and providing time for team and organization social activities as a non-mandatory activity. For example, using regular team meetings, “water cooler” type online informal chats, or just regular catch-ups. Employers should be proactive in establishing team-building social activities that can be carried out online to build team rapport. For new starters specifically, managers should seek to allow new team members to feel fully integrated and comfortable in their working role. This could be through arranging online social engagement opportunities (eg, short one-to-one introductory meetings with team members), and group activities to build rapport and social connection. Additionally, ensuring induction activities are adapted to the online context for new starters is important. These recommendations are in line with literature suggesting that managers should encourage team cohesion in order to protect the mental well-being of healthcare employees whilst working though infectious disease outbreaks.^30^

Findings relating to work-life balance were mixed, with many cited studies concluding that homeworking is beneficial for work-life balance, but others concluding conflict between work and family life as a key disadvantage associated with homeworking. Gaining a balance between work and home life whilst WFH was reported to be influenced by several factors (eg, job control, institutional setting, boundaries between work and home life)^15^^–^^17^ Therefore, it is recommended that after a prolonged period of WFH, employers and line managers should explore the feasibility of WFH for individual workers and their circumstances. For example, in terms of living situations or working preferences, to ensure that appropriate informed decisions can be made as to whether people go back to work, stay at home, or have a hybrid arrangement.

In terms of career impact, WFH was thought to impede career progression. Productivity literature also provided a mixed impact. Generally, productivity was found to increase when WFH, but was found to be impacted by human resources practices and dual role workers (eg, parents or carers). It is recommended that managers recognize the differences between WFH and office working and take account of this when role planning with their staff. Organizations and managers proactively showing support for employees is also recommended across various literature examining working through periods of trauma and disaster (eg,^30^^,^^31^ ). In the context of a pandemic invoking a transition to working from home in the future, a first step would be to ensure employees have suitable and designated time to discuss career progression and future goals and opportunities with managers to aid employee progression.

It is important to note that the fundamental suggestions being made here are not exclusive to homeworking (ie, they would also be recommended in office working environments). But, instead have been adapted for the remote working context and may require managers to be more proactive than usual and to ensure they have additional resources and guidance on hand. The majority of the themes reported in this review have mixed results (eg, autonomy, productivity, satisfaction, career prospects, work-life balance, general health, physical health, mental health). These findings indicate that WFH is a situation that differs greatly between individuals due to individual circumstances and contextual factors. This results in the need for a deeper understanding of the WFH context on a case-by-case basis, as managers need to understand they cannot simply give the same advice and guidance to all staff using a one-size-fits-all approach, as individual circumstances limit their application. For example, by engaging with their staff, managers can then identify what areas of work people might need advice or guidance for (eg, how to create a safe workspace, how to establish boundaries, how working hours could be adapted to aid other commitments such as caring responsibilities). Essentially, WFH requires more flexibility compared with office working, and requires forethought for making adaptations based on employees’ unique circumstances.

### 4.1. Limitations

The current review is, to our knowledge, the first umbrella review in the area of WFH collating findings from peer-reviewed review articles. However, there are several potential limitations to consider. Firstly, the definition of WFH has been described as a continuum,^32^ with many terms (eg, but not limited to, “homeworking,” “teleworking,” “remote working,” “telecommuting”) being used interchangeably (eg, discussed in reference 15). As the focus of this review was a “population of adult desk-based homeworking individuals,” all known terms covering the continuum of homeworking were incorporated in the search strategy (which can be found in Table S1); this resulted in reviews referring to both “telework” and “working from home” being included. As a result, slight differences in definitions could potentially contribute to confounds. Secondly, this umbrella review was mainly conducted by a single author (C.E.H.). To overcome this, findings were regularly discussed with the research team, a proportion of full-text screening was carried out by a second author (20%), and all listed authors contributed to the manuscript. Thirdly, searching for papers written in languages other than English, using additional databases, and including gray literature all may have resulted in a higher number of retained papers. Fourthly, due to a desire to include only systematic literature reviews (ie, to ensure systematically sound and thorough findings) there were potentially some narrative and rapid reviews removed at the full-text screening stage, which may have revealed additional barriers and facilitators to WFH (eg,^25^). However, the current review did collate a very thorough analysis of 19 factors captured under 3 key themes of “health,” “personal impact,” and “working environment.” Additionally, the literature search was carried out in June 2022 and the authors acknowledge that due to the fast pace associated with COVID-19-related research there may be additional insights available in more recently published literature. Lastly, the quality of the retained reviews ranged from critically low to high, with all reviews failing on certain quality appraisal criteria (eg, reporting on the sources of funding for included studies) and others failing on “critical” criteria (eg, risk of bias in individual studies) meaning that the current review’s results should be interpreted with caution.

### 4.2. Implications and future research

This umbrella review thematically analyzed the experience of WFH using peer-reviewed review articles. A total of 19 themes were derived from the literature, covering working environment, personal impact, and health. Many of the themes had conflicting research findings across the literature. Despite this, the current umbrella review has allowed for the formation of 8 recommendations for the future practice of WFH.

The mixed nature of the findings in this review suggest that more research is warranted into the experience of homeworking. Varied results could suggest that the findings are context specific and so further research is needed to understand the way the factors manifest in different contexts or with different workforces. In line with this, qualitative data providing rich insight into the experience of WFH in specific groups or occupations may result in more rigorous and supported findings.

## Author contributions

C.E.H., D.W., S.K.B. and N.G. conceptualized the review, created aims, and established inclusion criteria. C.E.H. conducted the database searches and all screening in accordance with the inclusion criteria alongside F.M. C.E.H. conducted quality appraisal of included papers. C.E.H. carried out the analysis, and C.E.H. drafted the initial manuscript; all authors provided critical revision of intellectual content. All authors approved the final manuscript.

## Supplementary material

Supplementary material is available at *Journal of Occupational Health* online.

## Funding

This study was funded by the National Institute for Health and Care Research Health Protection Research Unit (NIHR HPRU) in Emergency Preparedness and Response, a partnership between the UK Health Security Agency (UKHSA), King’s College London, and the University of East Anglia. The views expressed are those of the author(s) and not necessarily those of the NIHR, UKHSA, or the Department of Health and Social Care. For the purpose of open access, the author has applied a Creative Commons Attribution (CC-BY) licence to any Author Accepted Manuscript version arising.

## Conflicts of interest

The authors declare no potential conflicts of interest.

## Data availability

The datasets used and/or analyzed during the current study are available from the corresponding author on reasonable request.

## Supplementary Material

Web_Material_uiad013
