# Supplementary material for: Experiences of working from home: umbrella review
Source: J Occup Health. 2023 Dec 14;66(1):uiad013. doi: 10.1093/joccuh/uiad013 (PMC11020277; doi:10.1093/joccuh/uiad013)
Supplement: Web_Material_uiad013 [file web_material_uiad013.zip › Supplemental information v2.docx]

**Supplemental Information 1: Full Search strategy**

| **Concept** | **Combinations** | **Notes** | **Database: Ovid PsychINFO 1806 to January Week 3 2022** | | |
| --- | --- | --- | --- | --- | --- |
|  |  |  | **Search Number** | **Terms** | **Results** |
| **(1) Population / Context (Homeworkers)** | **Combine with OR** | Controlled vocabulary | 1 | exp Telecommuting/ | 556 |
|  |  | Free text (ab,ti = title and abstract) | 2 | (Work* adj3 home).ab,ti. | 5808 |
|  |  |  | 3 | WFH.ab,ti. | 18 |
|  |  |  | 4 | Flexible work*.ab,ti. | 1008 |
|  |  |  | 5 | Virtual work*.ab,ti. | 315 |
|  |  |  | 6 | Mobile work*.ab,ti. | 130 |
|  |  |  | 7 | Home based work*.ab,ti. | 66 |
|  |  |  | 8 | Remote employe*.ab,ti. | 31 |
|  |  |  | 9 | E-work*.ab,ti. | 97 |
|  |  |  | 10 | Home work*.ab,ti. | 558 |
|  |  |  | 11 | Homework*.ab,ti. | 5156 |
|  |  |  | 12 | Remote work*.ab,ti. | 275 |
|  |  |  | 13 | Telecommut*.ab,ti. | 313 |
|  |  |  | 14 | Telework*.ab,ti. | 395 |
|  |  |  |  | **AND** |  |
| **(2) Outcomes (Barriers and facilitators)** | **Combine with OR** | Free text (ab,ti = title and abstract) | 15 | Barrier*.ab,ti. | 83347 |
|  |  |  | 16 | Facilitator*.ab,ti. | 17517 |
|  |  |  | 17 | Benefit*.ab,ti. | 211157 |
|  |  |  | 18 | Consequence*.ab,ti. | 160224 |
|  |  |  | 19 | Difficult*.ab,ti. | 266740 |
|  |  |  | 20 | Experience*.ab,ti. | 707067 |
|  |  |  | 21 | Challeng*.ab,ti. | 277143 |
|  |  |  | 22 | Problem*.ab,ti. | 569077 |
|  |  |  | 23 | Issue*.ab,ti. | 418152 |
|  |  |  | 24 | Advantage*.ab,ti. | 82867 |
|  |  |  | 25 | Disadvantage*.ab,ti. | 35263 |
|  |  | Combined Population / Context terms | 26 | 1 + 2 + 3 + 4 + 5 + 6 + 7 + 8 + 9 + 10 + 11 + 12 + 13 + 14 | 13080 |
|  |  | Combined Outcome terms | 27 | 15 + 16 + 17 + 18 + 19 + 20 + 21 + 22 + 23 + 24 + 25 | 2046140 |
|  |  | Final search string (1 + 2) | 28 | 26 + 27 | 7474 |
|  |  | Final search string (1 + 2) Reviews | 29 | Limit 28 to reviews | **212** |

| **Concept** | **Combinations** | **Notes** | **Database: Ovid PsychINFO 1806 to January Week 3 2022** | | |
| --- | --- | --- | --- | --- | --- |
|  |  |  | **Search Number** | **Terms** | **Results** |
| **(1) Population / Context (Homeworkers)** | **Combine with OR** | Controlled vocabulary | 1 | exp Telecommuting/ | 1231 |
|  |  |  | 2 | exp Work from home/ | 521 |
|  |  | Free text (ab,ti = title and abstract) | 3 | (Work* adj3 home).ab,ti. | 9429 |
|  |  |  | 4 | WFH.ab,ti. | 674 |
|  |  |  | 5 | Flexible work*.ab,ti. | 968 |
|  |  |  | 6 | Virtual work*.ab,ti. | 351 |
|  |  |  | 7 | Mobile work*.ab,ti. | 173 |
|  |  |  | 8 | Home based work*.ab,ti. | 49 |
|  |  |  | 9 | Remote employe*.ab,ti. | 5 |
|  |  |  | 10 | E-work*.ab,ti. | 107 |
|  |  |  | 11 | Home work*.ab,ti. | 996 |
|  |  |  | 12 | Homework*.ab,ti. | 2706 |
|  |  |  | 13 | Remote work*.ab,ti. | 463 |
|  |  |  | 14 | Telecommut*.ab,ti. | 101 |
|  |  |  | 15 | Telework*.ab,ti. | 325 |
|  |  |  |  | **AND** |  |
| **(2) Outcomes (Barriers and facilitators)** | **Combine with OR** | Free text (ab,ti = title and abstract) | 16 | Barrier*.ab,ti. | 443059 |
|  |  |  | 17 | Facilitator*.ab,ti. | 41200 |
|  |  |  | 18 | Benefit*.ab,ti. | 1129463 |
|  |  |  | 19 | Consequence*.ab,ti. | 586636 |
|  |  |  | 20 | Difficult*.ab,ti. | 980074 |
|  |  |  | 21 | Experience*.ab,ti. | 1731460 |
|  |  |  | 22 | Challeng*.ab,ti. | 1290743 |
|  |  |  | 23 | Problem*.ab,ti. | 1415770 |
|  |  |  | 24 | Issue*.ab,ti. | 826648 |
|  |  |  | 25 | Advantage*.ab,ti. | 600957 |
|  |  |  | 26 | Disadvantage*.ab,ti. | 109128 |
|  |  | Combined Population / Context terms | 27 | 1 + 2 + 3 + 4 + 5 + 6 + 7 + 8 + 9 + 10 + 11 + 12 + 13 + 14 + 15 | 15384 |
|  |  | Combined Outcome terms | 28 | 16 + 17 + 18 + 19 + 20 + 21 + 22 + 23 + 24 + 25 + 26 | 7226392 |
|  |  | Final search string (1 + 2) | 29 | 27 + 28 | 7872 |
|  |  | Final search string (1 + 2) Reviews | 30 | Limit 29 to reviews | 580 |

| **Concept** | **Combinations** | **Notes** | **Database: Ovid PsychINFO 1806 to January Week 3 2022** | | |
| --- | --- | --- | --- | --- | --- |
|  |  |  | **Search Number** | **Terms** | **Results** |
| **(1) Population / Context (Homeworkers)** | **Combine with OR** | Free text (ab,ti = title and abstract) | 1 | (Work* adj3 home).ab,ti. | 7550 |
|  |  |  | 2 | WFH.ab,ti. | 290 |
|  |  |  | 3 | Flexible work*.ab,ti. | 822 |
|  |  |  | 4 | Virtual work*.ab,ti. | 341 |
|  |  |  | 5 | Mobile work*.ab,ti. | 155 |
|  |  |  | 6 | Home based work*.ab,ti. | 44 |
|  |  |  | 7 | Remote employe*.ab,ti. | 2 |
|  |  |  | 8 | E-work*.ab,ti. | 85 |
|  |  |  | 9 | Home work*.ab,ti. | 745 |
|  |  |  | 10 | Homework*.ab,ti. | 1953 |
|  |  |  | 11 | Remote work*.ab,ti. | 450 |
|  |  |  | 12 | Telecommut*.ab,ti. | 117 |
|  |  |  | 13 | Telework*.ab,ti. | 348 |
|  |  |  |  | **AND** |  |
| **(2) Outcomes (Barriers and facilitators)** | **Combine with OR** | Free text (ab,ti = title and abstract) | 14 | Barrier*.ab,ti. | 357515 |
|  |  |  | 15 | Facilitator*.ab,ti. | 33210 |
|  |  |  | 16 | Benefit*.ab,ti. | 801158 |
|  |  |  | 17 | Consequence*.ab,ti. | 467939 |
|  |  |  | 18 | Difficult*.ab,ti. | 723198 |
|  |  |  | 19 | Experience*.ab,ti. | 1247125 |
|  |  |  | 20 | Challeng*.ab,ti. | 1030151 |
|  |  |  | 21 | Problem*.ab,ti. | 1166312 |
|  |  |  | 22 | Issue*.ab,ti. | 642835 |
|  |  |  | 23 | Advantage*.ab,ti. | 486121 |
|  |  |  | 24 | Disadvantage*.ab,ti. | 88482 |
|  |  | Combined Population / Context terms | 25 | 1 + 2 + 3 + 4 + 5 + 6 + 7 + 8 + 9 + 10 + 11 + 12 + 13 | 11752 |
|  |  | Combined Outcome terms | 26 | 14 + 15 + 16 + 17 + 18 + 19 + 20 + 21 + 22 + 23 + 24 | 5624369 |
|  |  | Final search string (1 + 2) | 27 | 25 + 26 | 5801 |
|  |  | Final search string (1 + 2) Reviews | 28 | Limit 27 to reviews | **545** |

| **Concept** | **Combinations** | **Notes** | **Database: Ovid PsychINFO 1806 to January Week 3 2022** | | |
| --- | --- | --- | --- | --- | --- |
|  |  |  | **Search Number** | **Terms** | **Results** |
| **(1) Population / Context (Homeworkers)** | **Combine with OR** | Free text (ab,ti = title and abstract) | 1 | "Work* NEAR/3 home" | 16253 |
|  |  |  | 2 | WFH | 459 |
|  |  |  | 3 | "Flexible work*" | 3065 |
|  |  |  | 4 | "Virtual work*" | 4853 |
|  |  |  | 5 | "Mobile work*" | 1025 |
|  |  |  | 6 | "Home based work*" | 224 |
|  |  |  | 7 | "Remote employe*" | 28 |
|  |  |  | 8 | "E-work*" | 1593 |
|  |  |  | 9 | "Home work*" | 1446 |
|  |  |  | 10 | Homework* | 6862 |
|  |  |  | 11 | "Remote work*" | 1576 |
|  |  |  | 12 | Telecommut* | 945 |
|  |  |  | 13 | Telework* | 1905 |
|  |  |  |  | AND |  |
| **(2) Outcomes (Barriers and facilitators)** | **Combine with OR** | Free text (ab,ti = title and abstract) | 14 | Barrier* | 771414 |
|  |  |  | 15 | Facilitator* | 44223 |
|  |  |  | 16 | Benefit* | 1373333 |
|  |  |  | 17 | Consequence* | 901660 |
|  |  |  | 18 | Difficult* | 1314656 |
|  |  |  | 19 | Experience* | 2118486 |
|  |  |  | 20 | Challeng* | 2176175 |
|  |  |  | 21 | Problem* | 3953470 |
|  |  |  | 22 | Issue* | 1760340 |
|  |  |  | 23 | Advantage* | 1230911 |
|  |  |  | 24 | Disadvantage* | 185236 |
|  |  | Combined Population / Context terms | 25 | 1 + 2 + 3 + 4 + 5 + 6 + 7 + 8 + 9 + 10 + 11 + 12 + 13 | 355651 |
|  |  | Combined Outcome terms | 26 | 14 + 15 + 16 + 17 + 18 + 19 + 20 + 21 + 22 + 23 + 24 | 12557011 |
|  |  | Final search string (1 + 2) | 27 | 25 + 26 | 17543 |
|  |  | Final search string (1 + 2) Reviews | 28 | Limit 27 to reviews | **593** |

**Supplemental Information 2: Review characteristics**

**Please see Excel file**

**Supplemental Information 3: AMSTAR2**

AMSTAR-2 (A Measurement Tool to Assess Systematic Reviews) consists of the following 16 questions. ‘Critical questions’ are marked in bold text. Each item allows for the following response options: yes, partial yes, no. for the purpose of scoring, partial yes indicates a positive outcome.

1. Did the research questions and inclusion criteria for the review include the components of PICO?
2. **Did the report of the review contain an explicit statement that the review methods were established prior to the conduct of the review and did the report justify any significant deviations from the protocol?**
3. Did the review authors explain their selection of the study designs for inclusion in the review?
4. **Did the review authors use a comprehensive literature search strategy?**
5. Did the review authors perform study selection in duplicate?
6. Did the review authors perform data extraction in duplicate?
7. **Did the review authors provide a list of excluded studies and justify the exclusions?**
8. Did the review authors describe the included studies in adequate detail?
9. **Did the review authors use a satisfactory technique for assessing the risk of bias (RoB) in individual studies that were included in the review?**
10. Did the review authors report on the sources of funding for the studies included in the review?
11. **If meta-analysis was performed did the review authors use appropriate methods for statistical combination of results?**
12. If meta-analysis was performed, did the review authors assess the potential impact of RoB in individual studies on the results of the meta-analysis or other evidence synthesis?
13. **Did the review authors account for RoB in individual studies when interpreting/ discussing the results of the review?**
14. Did the review authors provide a satisfactory explanation for, and discussion of, any heterogeneity observed in the results of the review?
15. **If they performed quantitative synthesis did the review authors carry out an adequate investigation of publication bias (small study bias) and discuss its likely impact on the results of the review?**
16. Did the review authors report any potential sources of conflict of interest, including any funding they received for conducting the review?

AMSTAR-2 is not intended to be scored, and instead provides the following guidance for interpreting weaknesses:

- **High** - No or one non-critical weakness: the systematic review provides an accurate and comprehensive summary of the results of the available studies that address the question of interest;
- **Moderate** - More than one non-critical weakness: the systematic review has more than one weakness but no critical flaws. It may provide an accurate summary of the results of the available studies that were included in the review;
- **Low** - One critical flaw with or without non-critical weaknesses: the review has a critical flaw and may not provide an accurate and comprehensive summary of the available studies that address the question of interest;
- **Critically low** - More than one critical flaw with or without non-critical weaknesses: the review has more than one critical flaw and should not be relied on to provide an accurate and comprehensive summary of the available studies.

From: Shea BJ, Reeves BC, Wells G, Thuku M, Hamel C, Moran J, Moher D, Tugwell P, Welch V, Kristjansson E, Henry DA. AMSTAR 2: a critical appraisal tool for systematic reviews that include randomised or non-randomised studies of healthcare interventions, or both. BMJ 2017, 358, j4008.

| **Review** | **Q1** | **Q2** | **Q3** | **Q4** | **Q5** | **Q6** | **Q7** | **Q8** | **Q9** | **Q10** | **Q11** | **Q12** | **Q13** | **Q14** | **Q15** | **Q16** | **Rating** |
| --- | --- | --- | --- | --- | --- | --- | --- | --- | --- | --- | --- | --- | --- | --- | --- | --- | --- |
| Athanasiadou 2021 | Y | **P*** | Y | **Y** | N | N | **P°** | Y | **Y** | N | **N-MA** | N-MA | **N** | Y | **N-MA** | Y | Low |
| de Macedo 2020 | Y | **P*** | Y | **Y** | N | N | **P°** | Y | **N** | N | **N-MA** | N-MA | **N** | Y | **N-MA** | Y | Critically low |
| Di Fusco 2021 | N | **P*** | Y | **Y** | N | N | **P°** | N | **N** | N | **N-MA** | N-MA | **N** | Y | **N-MA** | Y | Critically low |
| Lunde 2022 | Y | **Y** | Y | **Y** | Y | Y | **Y** | Y | **Y** | N | **N-MA** | N-MA | **Y** | Y | **N-MA** | Y | High |
| Oakman 2020 | Y | **P*** | Y | **Y** | Y | Y | **Y** | Y | **Y** | N | **N-MA** | N-MA | **Y** | Y | **N-MA** | Y | High |
| Wutschert 2022 | Y | **P*** | Y | **Y** | Y | Y | **Y** | Y | **Y** | N | **N-MA** | N-MA | **Y** | Y | **N-MA** | Y | High |

**Table of AMSTAR-2 review appraisal results**

Note. Y indicates yes, P indicates partial yes, N indicates no. N-MA indicates non-meta analysis. P* indicates that a thorough design was provided, so received a partial score. P° indicates that a flow diagram was used alongside the number of excluded articles.
